# Resistance to the Bacteriocin Lcn972 Deciphered by Genome Sequencing

**DOI:** 10.3390/microorganisms11020501

**Published:** 2023-02-16

**Authors:** Susana Escobedo, Ana B. Campelo, Özgün C. O. Umu, María Jesús López-González, Ana Rodríguez, Dzung B. Diep, Beatriz Martínez

**Affiliations:** 1 Instituto de Productos Lacteos de Asturias (IPLA), CSIC, 33300 Villaviciosa, Spain; 2 Faculty of Chemistry, Biotechnology and Food Science, Norwegian University of Life Sciences, 1430 Ås, Norway; 3 Department of Paraclinical Sciences, Faculty of Veterinary Medicine, Norwegian University of Life Sciences, 1432 Ås, Norway

**Keywords:** *Lactococcus*, bacteriocin, resistance, adaptive evolution

## Abstract

In view of the current threat of antibiotic resistance, new antimicrobials with low risk of resistance development are demanded. Lcn972 is a lactococcal bacteriocin that inhibits septum formation by binding to the cell wall precursor lipid II in *Lactococcus*. It has a species-specific spectrum of activity, making Lcn972 an attractive template to develop or improve existing antibiotics. The aim of this work was to identify mutations present in the Lcn972-resistant clone *Lactococcus cremoris* D1-20, previously evolved from the sensitive strain *L. cremoris* MG1614. Whole-genome sequencing and comparison over the reference genome *L. cremoris* MG1363 identified several unexpected mutations in the parental strain MG1614, likely selected during in-house propagation. In the Lcn972R clone, two previously identified mutations were mapped and confirmed. Additionally, another transposition event deregulating cellobiose uptake was identified along with three point mutations of unknown consequences for Lcn972 resistance. Two new independent evolution experiments exposing *L. cremoris* MG1614 to Lcn972 revealed transposition of *IS*981 into the *LLMG_RS12285* locus as the predominant mutation selected by Lcn972. This event occurs early during evolution and was found in 100% of the evolved clones, while other mutations were not selected. Therefore, activation of *LLMG_RS12285* coding for a putative anti-ECF (extra-cytoplasmic function) sigma factor is regarded as the main Lcn972 resistance factor in *L. cremoris* MG1614.

## 1. Introduction

The growing threat of antibiotic resistance requires global action. Under the umbrella of the World Health Organization (WHO), different initiatives have fueled research to feed the preclinical and clinical pipelines with new antimicrobials as well as the implementation of non-traditional approaches to treat bacterial infections [1]. In this context, research on bacteriocins, gene-encoded antimicrobial peptides synthesized by bacteria, is shifting towards their applications in the clinical field. This shift is exemplified by the increasing number of reports on bacteriocin toxicity [2,3,4] and on their effectiveness in animal models (revised in [5]). Moreover, bacteriocins are seen not only as anti-infectives but also as potential microbiome-editing tools [6].

Bacteriocins comprise a large range of peptides with distinct structures and chemical properties that are translated into diverse modes of action, potency and spectra of inhibition that differ from those of current antibiotics. For instance, bacteriocins are often active against a discrete set of susceptible bacteria, overriding negative side effects on the endogenous microbiota [7]. Bacteriocins are also genetically amenable, providing novel templates for developing enhanced antimicrobials [8,9]. Regarding their modes of action, many bacteriocins from Gram-negative bacteria have intracellular targets, while bacteriocins from Gram-positive bacteria interfere mostly with cell envelope functions through disruptions in membrane permeability, pore formation and/or inhibition of cell wall synthesis (reviewed in [10,11]). Resistance to bacteriocins may compromise their prospects as anti-infectives and should be closely monitored, as cross-resistance with current antibiotics may concur [12,13]. Bacteriocin resistance is known to arise in laboratory settings but is also detected in natural environments [14]. Changes in the cell surface properties to reduce bacteriocin binding and pore formation, absence or modification of bacteriocin receptors, production of bacteriocin-degrading enzymes and multidrug ABC transporters are factors frequently involved in resistance to bacteriocins (reviewed in [15,16]).

Lactococcin 972 (Lcn972) is a non-pore-forming bacteriocin that inhibits cell wall biosynthesis by specifically binding to lipid II at the septum [17]. Lcn972 has a narrow spectrum of activity, targeting only *Lactococcus* species, and triggers the cell envelope stress response through the activation of the two-component system CesSR [18]. Lcn972 is not post-translationally modified, presents a well-defined ß-sandwich 3D structure in aqueous solutions and lacks the typical hydrophobicity shown by pore-forming bacteriocins [19]. Lcn972-like peptides appear to be widely spread in Firmicutes and Actinobacteria (InterPro IPR006540) and may play a relevant role in infection, as shown for *Listeria monocytogenes* [20]. These unique properties make Lcn972 an attractive template to develop or improve existing antibiotics. However, stable Lcn972-resistant (Lcn972R) mutants of laboratory and dairy lactococcal strains can be easily selected by sub-culturing in the presence of increasing Lcn972 concentrations [21,22]. The characterization of some of these Lcn972R clones revealed genetic reorganizations, an altered peptidoglycan composition and activation of antimicrobial peptide detoxification modules behind resistance to Lcn972 [12,21,23]. 

Adaptation of the plasmid-free laboratory strain *L. cremoris* MG1614 to Lcn972 resulted in the isolation of two Lcn972R clones: *L. cremoris* D1, which was highly resistant but unstable, and *L. cremoris* D1-20, with a stable Lcn972R phenotype that was isolated after sub-culturing *L. cremoris* D1 without selective pressure [21]. Transcriptomics of *L. cremoris* D1 revealed an activated CesSR response and transcriptional changes (mostly downregulation) in genes involved in carbohydrate metabolism, cell wall biosynthesis and others of unknown function [23]. A large chromosomal deletion (encompassing maltose metabolic genes, the TCS F and the phage infection Pip) and the presence of an insertion responsible for the transcriptional activation of *llmg2447* (LLMG_RS12285 in *L. cremoris* MG1363, Accession NC_009004.1), which encodes a putative extra-cytoplasmic function (ECF) anti-sigma factor, were detected in both Lcn972R clones. Expression of *llmg2447* led to Lcn972 resistance but below the levels shown by *L. cremoris* D1 and D1-20, anticipating the role of other factors in resistance [23].

In this work, we aimed to gain deeper insight into the possible mutations behind resistance to Lcn972 and the genome sequences of *L. cremoris* MG1614, and the Lcn972R clone D1-20 were determined by next-generation sequencing. The results uncovered unexpected mutations in the parental strain, confirmed the occurrence of the chromosomal deletion and mobilization of *IS*981 identified previously and untapped other mutations present in the Lcn972R clone. Evolution experiments were also replicated to follow the acquisition of some of these mutations during exposure to Lcn972 and after releasing the stress.

## 2. Material and Methods

### 2.1. Bacterial Strains and Culture Conditions

*Lactococcus cremoris* MG1614 [24] and its Lcn972R evolved clone *L. cremoris* D1-20 [21] were routinely grown in M17 (Formedium, Norfolk, UK) supplemented with glucose at 0.5% at 30 °C. Growth on maltose was determined in Bromocresol Purple maltose broth BCP-mal (w/v): 0.5% tryptone, 0.3% meat extract, 0.5% maltose and 0.004% bromocresol purple. When needed, Lcn972 was added at the indicated concentrations. Purified Lcn972 stock was in 50 mM sodium phosphate buffer, pH 6.8, with a specific activity of at 39.4 Arbitrary units (AUs)/µg (25,600 AU/mL, 650 µg/mL).

### 2.2. Library Preparation and Sequencing

Chromosomal DNA was isolated with the Qiagen DNeasy Blood & Tissue Kit (Qiagen, Hilden, Germany), concentration was measured by Qubit using Qubit^®^ dsDNA HS Assay Kit (Invitrogen, Waltham, MA, USA) and quality was checked via gel electrophoresis (1% agarose gel). The concentration of DNA from each sample was diluted to 0.2 ng/µL. Sequencing was performed in Illumina MiSeq platform and preparation was conducted as described in Nextera XT DNA sample preparation guide (https://support.illumina.com/downloads/nextera_xt_sample_preparation_guide_15031942.html, accessed on 12 April 2022). Briefly, Nextera XT DNA kit (Illumina, San Diego, CA, USA) was used for the fragmentation of the genomic DNA. PCR was carried out for sample-specific dual indexing using the Nextera XT Index kit (Illumina, San Diego, CA USA) that contains index primers with 8-base indices adjacent to the P5 or P7. Cleaning up of indexing PCR products was performed using AMPure XP beads (Beckman Coulter Genomics, USA), where the amplicon size in the pool was chosen to be >500 bp. The pool of the normalized libraries was denatured prior to loading of samples into the MiSeq instrument. The sequencing was performed using MiSeq v3 reagent kit (Illumina, San Diego, CA USA). Reads were subjected to quality analysis with FastQC v0.11.9 [25], and those with Phred scores of less than 30 were removed. Sequence reads were assembled with SPAdes 3.1.0 [26]. Assemblies with different k-mer sizes were compared, and the best one with 131 contigs and an N_50_ value of  59,995 bp for MG1614 and 123 contigs and N_50_ of 57,818 bp for D1_20 were selected for scaffolding. The average sequencing coverage was estimated to be 50-fold. CONTIGuator _V2.7 and Mauve 2.4.0 software tools [27,28] were used to resolve the relative position of the contigs obtained over *L. cremoris* MG1363 genome (GenBank accession NC_009004.1) used as the reference. Determination of single-nucleotide polymorphisms (SNPs) was performed using 0.37.0 version of the Breseq computational pipeline [29]. Rapid Annotation Subsystem Technology (RAST) server [30] was used for initial automatic genome annotation.

The draft genomes of MG1614 and D1-20 strains were deposited in the NCBI GenBank database with accession numbers JAPZLG010000000 and JAPZLH000000000, respectively.

### 2.3. RNA Extraction and Quantitative Reverse Transcription-PCR (RT-qPCR)

Two independent cultures of *L. cremoris* MG1614 and the mutant *L. cremoris* D1-20 were inoculated at 1% in GM17 broth and grown at 30 °C until they reached an OD_600_ of 1.0, when RNAprotect Bacteria Reagent (Qiagen) was added. Total RNA was extracted using the illustra RNAspin Mini RNA Isolation Kit (GE Healthcare, Chicago, IL, USA) and treated with SUPERase RNase Inhibitor (Ambion) and Turbo DNase (Ambion). RNA concentration was determined by absorbance at 260 nm in an Epoch microplate spectrophotometer (BioTek) and its quality was checked by agarose gel electrophoresis. One microgram of each RNA sample was used to generate cDNA with the iScript cDNA Synthesis Kit (Bio-Rad). RT-qPCR was performed in a 7500 Fast Real-Time PCR System (Applied Biosystems). Primers used for the amplification are listed in Table S1 and were supplied by Macrogen. Amplification was carried out in 25 μL containing 0.005 μg cDNA, 1× Power SYBR Green (Applied Biosystems) and each primer at a concentration of 0.56 µM. After incubation at 95 °C for 10 min, amplification proceeded with 40 cycles of 95 °C for 15 s and 60 °C for 1 min. Fold changes were calculated following the 2^−ΔΔCt^ method [31], and the reference gene was the elongation factor Tu *tuf*.

### 2.4. Evolution Experiments

*L. cremoris* MG1614 was adapted to grow in the presence of increasing Lcn972 concentrations, as previously described [21], with some modifications. Two isolated colonies (A, B) were inoculated in 2 mL of GM17 and grown overnight (T0). Adaptive evolution started by diluting 1:100 these T0 cultures in 2 mL GM17 with 10 AU/mL Lcn972 and further incubation for 24 h (T1). Transfers were subsequently conducted in 20 (T2), 40 (T3), 80 (T4), 160 (T5), 640 (T6) and 1280 AU/mL Lcn972 (T7). Two control experiments (MA and MB) were identically carried out without Lcn972. Samples from T4 (80 AU/mL) and T7 (1280 AU/mL) and from the control experiments (MA and MB after 7 transfers) were spread on GM17 plates. Single colonies (n = 31 per sample) were inoculated in two deep-well microtiter plates (500 µL GM17 per well) and grown for 24 h. *L. lactis* MG1614 and D1-20 were also inoculated along with a blank well (only GM17) as negative control. These master plates were further used for phenotypic testing, as described below. Evolved clones from samples T7A and T7B along with *L. cremoris* MG1614 and D1-20 as controls were inoculated (5 µL) in a deep-well microtiter plate (0.5 mL GM17) to proceed with successive transfers in the absence of Lcn972 (stabilization). Up to eleven transfers were carried out accounting for approximately 80 generations. These stabilized clones were also subjected to phenotypic testing.

### 2.5. Phenotypic Testing during Evolution and Stabilization

The cultures from the deep-well master plates were diluted 1/4 into a 96-microtiter plate (200 µL GM17). Resistance to Lcn972 was checked by inoculating with 5 µL from this 1/4 dilution into 200 µL GM17 supplemented with 80 AU/mL Lcn972. Growth was assessed visually by the presence of cell pellets after 24 h incubation at 30 °C and by measuring OD600 in a microtiter reader Benchmark Plus microplate spectrophotometer (Bio-Rad, Hercules, CA, USA). The same inoculum was used in 96-well microtiter plates with 200 µL BCP-mal to determine fermentation by color change (purple to yellow) after 24 h incubation at 30 °C.

### 2.6. PCR Detection of IS905::celB and IS981::LLMG_RS12285

DNA extracts were prepared from cultures grown for 24 h at 30 °C. Thus, 100 µL was transferred to a 96-well PCR plate (Applied Biosystems). After centrifugation at 3434× *g* for 15 min at 4 °C, pelleted cells were boiled (5 min, 95 °C) in 20 µL lysis buffer (0.25% SDS, 50 mM NaOH). Ultra-pure water (180 µL) was added and the supernatant collected after centrifugation. PCR reactions were carried out with Taq DNA Polymerase 2.0 x Master Mix Red (Ampliqon, Ampliqon Denmark) in a final volume 12.5 µL and 1 µL of each DNA extract. Primers are described in Table S1. PCR conditions were denaturation at 95°C for 4 min; 30 cycles at 95°C for 30 s, 50°C for 30 s and 72°C for 2 min; and a final extension step of 72°C for 7 min. Sanger sequencing of *IS*905::*celB* PCR product was carried at Macrogen.

### 2.7. MIC Determinations

Lcn972 minimal inhibitory concentrations (MICs) were determined by the broth microdilution method, as previously described [17].

### 2.8. Microscopy

Phase contrast images of lactococcal cells from overnight cultures in GM17 were observed with a 100× phase contrast objective lens in a DMi8 (Leica) microscope equipped with a Leica DFC365FX camera.

## 3. Results and Discussion

### 3.1. Genome Analysis of L. lactis MG1614 Reveals Laboratory-Selected Mutations

*L. cremoris* MG1614 is a spontaneous rifampicin- and streptomycin-resistant mutant of *L. lactis* MG1363 generated by Gasson in 1983 [24]. This strain has been routinely used in conjugation experiments, as a phage host [32], and as an indicator strain for the bacteriocin Lcn972 [33]. The genome of *L. lactis* MG1614 was sequenced with an average coverage of 50× and assembled in 131 contigs. It was estimated to be 2,437,439 bp, and 2360 coding sequences.

When mapped and compared to its parent *L. lactis* MG1363, the contigs covered 93% of the chromosome. We were able to detect 22 mutations, including single-nucleotide polymorphisms (SNPs) and small deletions and insertions (INDELS), between MG1614 and the reference strain (Table 1). Three of these mutations are located in intergenic regions; the other two affect genes codifying for transcriptional regulators, while the rest fall on genes involved in sugar metabolism, DNA repair and several hypothetical proteins (Table 1). Interestingly, mutations involved in resistance to rifampicin and streptomycin were detected. Resistance to rifampicin (Rif^r^) is likely due to the G→A mutation at position 1,968,674 (Table 1), as mutations in the *rpoB* gene, which encodes the β subunit of the RNA polymerase, decrease the affinity of RNAP for rifampicin [34]. Likewise, chromosomally acquired streptomycin resistance is frequently linked to mutations in the gene encoding the ribosomal protein S12 (*rpsL*) [35], such as the T→C mutation found at position 2,517,498 in *L. cremoris* MG1614 (Table 1).

**Figure 1:**
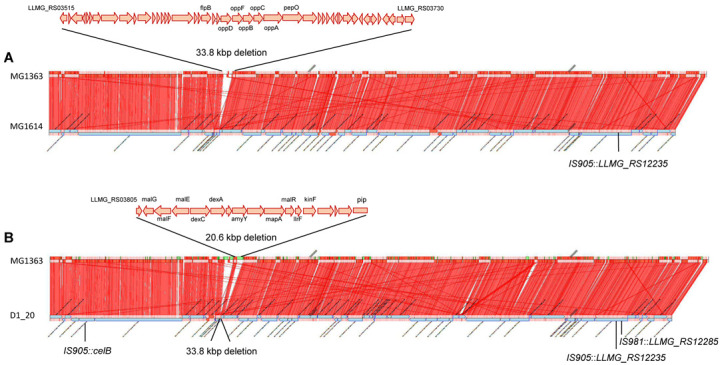
Synteny graphic generated by CONTIGuator showing *L. cremoris* MG1363 reference genome on top and assembled contigs of *L. cremoris* MG1614 (**A**) and the Lcn972-resistant mutant D1-20 (**B**) on the bottom. Genome rearrangements, such as deletions and insertions, are shown. Location and schematic representation of the open reading frames (ORFs) in the deleted sections are indicated.

In addition to point mutations, we also noticed the insertion of a copy of IS*905* into *LLMG_RS12235* (pseudo) and a 33.8 kbp deletion encompassing, among others, the transcriptional regulator FNR-like protein B encoded by *flpB* involved in Zn^+^ homeostasis and genes coding for the oligopeptide transport system (Opp) (Figure 1A). The deleted region is flanked by transposases Tnp1297 and Tnp981, which might have been involved in intra-chromosomal rearrangements. Nevertheless, several transposition functions are also encoded within the deleted DNA, which could have participated as well. This deletion appears to have been selected during propagation of this strain in our laboratory because it was not detected by PCR in *L. lactis* MG1614 from older strain repositories (e.g., *L. lactis* MG1614.2, 32) or in other MG1363-derived strains, such as NZ9000 and their original strain *L. cremoris* NCDO712 (Figure S1).

The accumulation of mutations in related lactococcal strains has already been shown to occur, resulting in, for example, different carbohydrate fermentation patterns [36,37]. For *L. cremoris* MG1614, a preliminary insight into its fermentation profile did not reveal major changes compared to its ancestor (Table S2). Nevertheless, the large deletion and other mutations that might alter protein function shown in Table 1 deserve further attention, considering that strains such as MG1614 are used worldwide as model lactic acid bacteria for genetic and physiological studies.

### 3.2. Genome Analysis of the Lcn972R Mutant L. lactis D1-20

The genome of *L. cremoris* D1-20 was represented by 123 contigs with a 97.9% coverage compared to the reference *L. cremoris* MG1363, and when the nucleotide sequence was aligned, breseq analysis revealed three point mutations that were exclusively found in D1-20 (Table 1). The conservative amino acid change L633I in the ribonucleoside–thriphosphate reductase (*nrdD)* that catalyzes the reductive synthesis of deoxyribonucleotides from their corresponding ribonucleotides (InterPro IPR012833) is not likely to impair its function and, thus, has no role in resistance to Lcn972.

The second mutation was found in the putative promoter of LLMG_RS08235 encoding a putative ABC transporter ATP-binding protein. Several ABC transporters are involved in resistance to antimicrobial peptides [38] but this mutation was not studied any further. The location of the nucleotide change (G→A, complementary strand) minimally modifies the extended −10 sequence from TGATATAAT in MG1614 to TAATATAAT in D1-20. Moreover, in previous transcriptional analyses of *L. cremoris* D1 (from which, D1-20 was isolated after growth without Lcn972), no significant changes in expression were observed for this gene [23].

The third mutation was found in the putative aspartate protease *LLMG_RS11020* with a 3D (Asp-Asp-Asp) domain and it was confirmed by Sanger sequencing. It created a truncated protein lacking the 3D domain. This domain is found in MltA-like lytic transglycosylases and other peptidoglycan remodeling proteins with putative O-glycosyl hydrolase activity (InterPro IPR010611). Based on this, an insertional mutant was generated in *L. cremoris* MG1363 but only a slight increase in the Lcn972 MIC was noted (40 vs. 20 AU/mL). Thus, we presume that a defective *LLMG_RS11020* does not confer resistance to Lcn972.

In addition to these mutations, two previously identified chromosomal rearrangements were confirmed, as shown in Figure 1B. On one hand, the 20.6 kbp deletion that encompasses genes involved in maltose metabolism, the two-component system (TCS) F and the 5′ end of phage receptor protein gene *pip,* have been proved to be responsible for both the impaired growth on maltose and the phage-resistant phenotype shown by *L. cremoris* D1-20 [21]. On the other hand, the insertion of IS*981* into the *LLMG_RS12285* locus leads to overexpression of this putative ECF anti-sigma factor and resistance to Lcn972 [23]. There was another mobilization event located in the *celB* cluster (described below), reinforcing the role of insertion sequences as driving forces in stress adaptation and evolvability in bacteria, and in lactococci in particular [39,40].

### 3.3. Cellobiose Uptake Is Activated in L. cremoris D1-20

An insertion of a copy of the IS*905* element upstream of the *LLMG_RS00985-celB* gene cluster was detected in *L. cremoris* D1-20. CelB is the IIC component of the cellobiose phosphoenolpyruvate-dependent phosphotransferase system (PTS) that is transcriptionally coupled to *LLMG_RS00985* of unknown function [41,42]. Downregulation of cellobiose metabolism has been linked to tolerance to Lcn972 in Lcn972-producing lactococci [42]. Therefore, the impact of the mobilization of *IS*905 into this locus (*IS*905::*celB*) was studied further.

**Figure 2:**
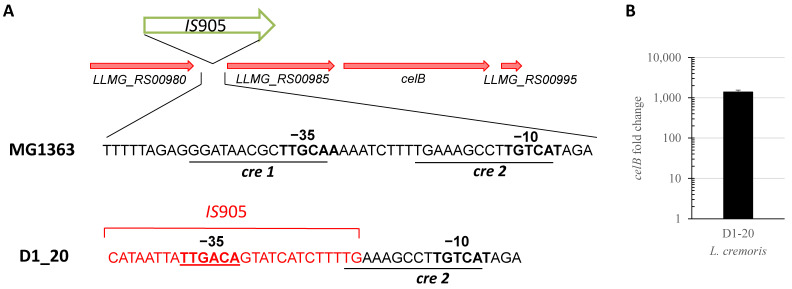
Schematic representation of the cellobiose cluster and nucleotide sequence of the region upstream of *LLMG_RS00985-celB* genes spanning the *cel* promoter of *L. cremoris* MG1363, and the newly created promoter after integration of *IS*905 in the Lcn972-resistant mutant D1-20 (**A**). The −35 and −10 elements are shown in bold, red was used for DNA incorporated from *IS*905, while *cre* sites 1 and 2 are underlined. (**B**) *celB* expression in exponentially growing *L. cremoris* D1-20 in GM17 compared to *L. cremoris* MG1614 as determined by RT-qPCR.

PCR and sanger sequencing using primers P0186F and P0186R (Table S1) verified the insertion of *IS*905 and detected an 8 bp duplication ATCTTTTG at both sides of the insertion site located between the −35 and −10 elements of the promoter of this cluster. This insertion *IS*905::*celB* (Figure 2A) places a canonical −35 (TTGACA) at 20 nt of the original −10 element. It is also worth noting that although duplication of the ATCTTTTG octanucleotide keeps the putative catabolite binding element *cre*2, the other repressor binding motif *cre*1 is not present in the newly created promoter. To confirm if the novel −35 region could create a functional promoter and activate transcription of the *celB* cluster in spite of growth on glucose (alternative sugars are subject to carbon catabolite repression), the expression levels of *celB* were quantified by RT-qPCR. Compared to *L. cremoris* MG1614, *celB* expression in *L. cremoris* D1-20 was three orders of magnitude higher (Figure 2B). In addition, contrary to its ancestor, *L. cremoris* D1-20 was able to ferment cellobiose (Table S2), confirming the activation of cellobiose metabolism.

This result opposes the above-mentioned role of downregulation of cellobiose uptake in tolerance to Lcn972 [42]. However, this insertion event might have occurred during the propagation of *L. cremoris* D1 in the absence of Lcn972 that preceded the isolation of *L. cremoris* D1-20. *celB* was one of the repressed genes in *L. cremoris* D1 [23] and the *IS9*905::*celB* mutation could have been selected as a countermeasure. Activation of cellobiose metabolism appears to occur frequently in laboratory strains derived from *L. cremoris* MG1363 in multiple ways from single-nucleotide mutations in promoter regions to disruption of a transcriptional repressor [36,37,43].

### 3.4. Transposition of IS981 into the LLMG_RS12285 Promoter Is Selected Early during Evolution in the Presence of Lcn972 and Is Not Lost upon Successive Cultivation without Selective Pressure

In view of the transposition events detected by genome sequencing in *L. cremoris* D1-20, two independent evolution experiments (A and B), growing *L. cremoris* MG1614 in the presence of Lcn972, were carried out to follow the acquisition of selected mutations, namely maltose fermentation and the insertions *IS*905::*celB* and *IS*981::*LLMG_RS12285*. Two control experiments without Lcn972 (MA, MB) were also carried out to determine if any of these mutations could occur simply after sequential sub-culturing, i.e., without selective pressure. Samples after growth at 80 AU/mL Lcn972 (T4A, T4B) and the final transfer into 1280 AU/mL Lcn972 (T7A, T7B) were withdrawn and plated on GM17. From the control experiment, the sample was taken after seven transfers. Doubling Lcn972 concentrations from 10 AU/mL allowed for full growth in 24 h until 1280 AU/mL, where CFU counts were 0.5 (T7A) and 1.0 (T7B) logCFU units lower than the control experiments (MA, MB) without Lcn972 (Table 2). Notably, pinpoint colonies were observed along with control-size colonies in the GM17 plates from T7A but not from T7B samples (Figure S2). Regardless of the colony phenotype, a total of 31 single colonies from each replicate of T4 (80 AU/mL), T7 (1280 AU/mL) and control (MA and MB) were screened (Table 2). Contrary to D1-20, all the newly evolved clones were able to grow in BCP-mal and lacked the transposition *IS*905::*celB*. Instead, all clones carried the insertion of *IS*981 into the *LLMG_RS12285* locus, pointing to this transposition event as the most frequent during evolution that occurs, at least after growth in Lcn972 at 80 AU/mL (T4 samples).

**Figure 3:**
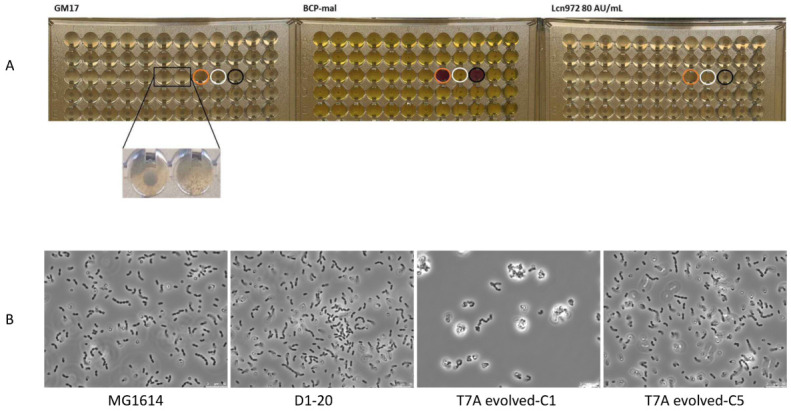
Phenotypic testing of evolved clones at the end of the evolution experiment. (**A**) Growth in GM17, BCP-mal for maltose fermentation and GM17 supplemented with Lcn972 at 80 AU/mL. Each well was inoculated with isolated clones after growth for 80 generations without Lcn972. The circles frame control wells: uninoculated (only broth, orange), *L. cremoris* MG1614 (white) and *L. cremoris* D1-20 (black). The insert displays the aggregation phenotype shown by some of the evolved clones. (**B**) Phase contrast microscopy of overnight GM17 cultures of the ancestor *L. cremoris* MG1614, the Lcn972-resistant clone D1-20 and two representative evolved clones from the T7A sample showing an aggregating (C1) or a typical lactococcal phenotype (C5).

The evolved clones from samples T7A (n = 31) and T7B (n = 26) were sequentially transferred to GM17 for 80 generations to assess the stability of the Lcn972R phenotype, the ability to grow on maltose and the insertion *IS*981::*LLMG_RS12285* without selective pressure*. IS*905::*celB* was also checked to determine if it could take place during subsequent transfers. All the clones retained the same features as at the starting point, that is: they were able to growth on maltose and in Lcn972 at 80 AU/mL (Figure 3), and *IS*905::*celB* was not detected (Table 2). Of note, the mobilization of *IS*981 into the *LLMG_RS12285* locus was not lost, supporting the notion that this transposition event is stable. Thus, the replication of the evolution experiment confirmed that the 20.6 kbp deletion and the mutation *IS*905::*celB* seem to be unique to *L. cremoris* D1-20.

Following stabilization, it was observed that the 24 h cultures in GM17 of one-third (n = 10) of the evolved clones from T7A reached ODs below 50% of that of MG1614 and D1-20. These clones did not grow homogenously and produced a clumpy pellet, clearly distinct from the compact pellet of D1-20 (see insert in Figure 3A). To better appreciate the morphological changes, two representative clones with a clumpy phenotype (C1) or with a compact pellet (C5) were observed under the microscope (Figure 3B). The slow-growth variant formed clumps and twisted chains, a phenotype neither observed in the other Lcn972R clone from the same evolution experiment nor in *L. cremoris* MG1614 and D1-20. Regardless of this phenotype, the MIC of Lcn972 for these two clones was over 160 AU/mL, confirming its adaptation to Lcn972. Cell aggregation and a clumping phenotype were previously correlated with alterations in cell wall components such as the lack of the polysaccharidic pellicle that covers the lactococcal cells [44]. Importantly, this pellicle has been recognized as the receptor for many lactococcal phages [45,46], suggesting the possibility that adaptation to Lcn972 may also select for cross-resistance to phages, a phenotype that may deserve further attention. An altered surface has been frequently linked to bacteriocin resistance in lactococci, regardless of the specific mode of action [47,48,49], and Lcn972 is not an exception to it.

Finally, it is worth noting that these slow-growth variants were only detected in clones from T7A and not in its replicate T7B. This result underpins the concomitant selection of other mutations and reflects the heterogeneity in the mutational landscape within a bacterial population [50,51].

## 4. Conclusions

The results of this work remind us about the multiple mutations that can be unintentionally selected in model bacteria, as shown here for our *L. cremoris* MG1614. The underlying mutations found in *L. cremoris* D1-20 were identified and, on the basis of the results from the replication experiment, the *IS*981::*LLMG_RS12285* mutation is proposed as the main Lcn972 resistance factor in *L. cremoris* MG1614, while other mutations appear to be unique to this particular clone. Overall, these results also highlight the difficulties encountered for predicting the outcome of evolution experiments based on single-clone analyses.

## Figures and Tables

**Table 1:** Point mutations in *L. cremoris* MG1614 and D1_20 vs. MG1363 (Accession NC_009004).

Position	Change	Annotation	Locus_tag	Description	MG1614	D1_20
218,269	+C	coding (1042/1056 nt)	LLMG_RS01195→	DUF2142 domain-containing protein	*	*
269,158	C→A	L633I (CTT→ATT)	LLMG_RS01465→	Anaerobic ribonucleoside-triphosphate reductase	-	*
446,872	C→A	intergenic (−46/−65)	LLMG_RS02295←/→LLMG_RS02300	trehalose operon repressor/PTS sugar transporter subunit IIA	*	-
482,337	C→T	H723Y (CAT→TAT)	LLMG_RS02480→	glycoside hydrolase family 65 protein	*	*
636,592	(A)_7→6_	coding (102/969 nt)	LLMG_RS03345←	ABC transporter permease	*	*
643,970	T→G	T7P (ACT→CCT)	LLMG_RS03375←	IS3 family transposase	*	*
894,627	+G	pseudogene (1075/1176 nt)	LLMG_RS04715→	cell surface protein	*	*
945,239	+C	coding (307/330 nt)	LLMG_RS04990→	hypothetical protein	*	*
1,093,299	+C	coding (3979/4050 nt)	LLMG_RS13005→	cell wall anchor	*	*
1,093,562	+C	pseudogene (175/839 nt)	LLMG_RS13155→	hypothetical protein	*	*
1,134,220	C→A	T147K (ACA→AAA)	LLMG_RS05920→	alpha-glucuronidase	*	*
1,210,283	(A)_5→6_	pseudogene (515/1046 nt)	LLMG_RS06265←	LacI family transcriptional regulator	*	*
1,223,557	2 bp→AG	coding (236-237/237 nt)	LLMG_RS13200→	hypothetical protein	*	*
1,318,548	(A)_7→6_	pseudogene (98/412 nt)	LLMG_RS06810→	hypothetical protein	*	*
1,612,853	C→A	intergenic (-60/+63)	LLMG_RS08235←/←LLMG_RS08240	ABC transporter ATP-binding protein/EamA family transporter	-	*
1,614,130	+T	coding (1149/1278 nt)	LLMG_RS08245←	citrate:sodium symporter	*	*
1,660,056	(T)_7→8_	intergenic (-14/+100)	LLMG_RS08475←/←LLMG_RS08480	hypothetical protein/DNA repair protein RecN	*	*
1,826,465	(T)_6→7_	intergenic (-23/+300)	LLMG_RS09240←/←LLMG_RS09245	metal-dependent hydrolase/cold-shock protein	*	*
1,968,674	G→A	S491F (TCT→TTT)	LLMG_RS09935←	DNA-directed RNA polymerase subunit beta	*	*
2,112,674	+T	pseudogene (396/1083 nt)	LLMG_RS10765←	hypothetical protein	*	*
2,159,055	G→A	R110 * (CGA→TGA)	LLMG_RS11020←	aspartate protease	-	*
2,183,058	(A)_5→4_	coding (399/1161 nt)	LLMG_RS11130←	helix-turn-helix domain-containing protein	*	*
2,191,553	G→A	A94T (GCT→ACT)	LLMG_RS11180→	DNA replication and repair protein RecF	*	*
2,266,855	C→T	R54W (CGG→TGG)	LLMG_RS11605→	arginine repressor	*	*
2,517,498	T→C	K56R (AAA→AGA)	LLMG_RS12900←	30S ribosomal protein S12	*	*

→, Nucleotide changes; * Mutation present.

**Table 2:** Frequency of selected mutations during evolution in the presence of Lcn972.

Sample	Lcn972 (AU/mL)	Log CFU/mL	n ^1^	Growth on Maltose ^2^	Growth in Lcn972 (80 AU/mL) ^2^	*IS*905 Insertion ^2^	*IS*981 Insertion ^2^
MA	0	8.85	31	31	0	0	0
MB	0	8.91	31	31	0	0	0
T4A	80	8.93	31	31	28	0	31
T4B	80	8.30	31	31	31	0	31
T7A	1280	8.42	31	31 (31)	31 (31)	0	31
T7B	1280	7.86	31	31 (26)	31 (26)	0 (0)	31 (26)

^1^ n, number of colonies tested; ^2^ positive clones; numbers in brackets are positive clones after growth for 80 generations without Lcn972. For T7B, 26 clones were subjected to stabilization.

## Data Availability

All the generated data are included in the Results section and in Supplementary Material. Genome sequence of *L. cremoris* MG1614 and the Lcn972R mutant *L. cremoris* D1-20 were deposited in the NCBI GenBank database under the accession numbers JAPZLG000000000 and JAPZLH000000000, respectively.

## Supplementary Materials

The following supporting information can be downloaded at: https://www.mdpi.com/article/10.3390/microorganisms11020501/s1, Table S1. Primers used in this work; Table S2. Fermentation pattern of *Lactococcus cremoris* MG1363, MG1614 and the Lcn972R clone D1-20. Figure S1. PCR detection of the 33.8 kbp deletion present in *Lactococcus cremoris* MG1614; Figure S2. Presence of pinpoint colonies in samples taken at the end of the evolution experiment.
