# Supplementary material for: Resistance to the Bacteriocin Lcn972 Deciphered by Genome Sequencing
Source: Microorganisms. 2023 Feb 16;11(2):501. doi: 10.3390/microorganisms11020501 (PMC9964109; doi:10.3390/microorganisms11020501)

Table S1. Primers used in this work

Name	Sequence (5´-3´)	Position*	Reference
**FDF**	GCTCAACCATCATTTCATCTGA	LLMG_RS03515 (nt 669574)	This work
**FDR**	TTAGCTGTTACTCTGCTTCCATC	LLMG_RS03720 (nt 703837)	
**Llmg2437UP**	ATAGGTTCACTATCATAATA	LLMG_RS12235 (nt 2388181)	This work
**Llmg2437DW**	ATAAGAACAGGCATGTTGGC	LLMG_RS12235 (nt 2388608)	
**P0186F**	GCAAGCTCGCATCGTTAATC	LLMG_RS00980 (nt 174386)	This work
**P186R**	TCCACCATCACTTCCATTCC	LLMG_RS00980 (nt 175132)	
**0734F**	GAAATGGCCCTGCGACGTGTAG	LLMG_RS03790 (nt 716407)	Roces et al., 2012a
**0752R**	CGCTGCATCCAATGTCACAGTC	LLMG_RS03885 (nt 741049)	
**Tuf-F**	GGTAGTTGTCGAAGAATGGAGTGTGA	tuf (reference) (nt 2033,958).	Campelo et al., 2011
**Tuf-R**	TAAACCAGGTTCAATCACTCCACACA	tuf (reference) (nt 2033983)	Campelo et al., 2011
**celB-qF**	ATTTGGCCCGTGCTTACG	LLMG_RS00985 (nt 176464)	Campelo et al., 2011
**celB-qR**	TTTGGCAAACCTGCAAATAGG	LLMG_RS00985 (nt 176521)	Campelo et al., 2011
**X1**	TTTTGTCTGAATTCCAAAACTCTT	LLMG_ RS12275 (nt 2398620)	Roces et al., 2012b
**X3**	TGTTCGATACATAAAAGCCCG	LLMG_RS12280 (nt 2398989)	Roces et al., 2012b

*Accession MG1363: NC_009004.1

References

Campelo AB, Gaspar P, Roces C, Rodríguez A, Kok J, Kuipers OP, Neves AR, Martínez B. 2011. The Lcn972 bacteriocin-encoding plasmid pBL1 impairs cellobiose metabolism in *Lactococcus lactis*. *Appl Environ Microbiol*. 77(21):7576-7585. doi: 10.1128/AEM.06107-11

Roces C., Courtin P., Kulakauskas S., Rodríguez A., Chapot-Chartier M. P., Martínez B. (2012a). Isolation of *Lactococcus lactis* mutants simultaneously resistant to the cell wall-active bacteriocin Lcn972, lysozyme, nisin and bacteriophage c2. *Appl. Environ. Microbiol*. 78 4157–4163. doi: 10.1128/AEM.00795-12

Roces C., Pérez V., Campelo A. B., Blanco D., Kok J., Kuipers O. P., et al. (2012b). The putative lactococcal extracytoplasmic function anti-sigma factor *llmg2447* determines resistance to the cell wall-active bacteriocin lcn972. *Antimicrob. Agents Chemother.* 56:5520–5527. doi: 10.1128/AAC.01206-12

Table S2. Fermentation pattern of *Lactococcus cremoris* MG1363, MG1614 and the Lcn972R clone D1-20 determined by the commercial PhenePlate system (Bactus, Stockholm, Sweden) as recommended by the supplier. Shadowed cells indicate fermentation (+). w, weak reaction.

	**MG1614**	**D1-20**	**MG1363**
**Arabinose**			
**Xylose**			
**Galactose**	w	w	+
**Maltose**	+		+
**Cellobiose**		+	
**Trehalose**	+	+	+
**Palatinose**			
**Sucrose**			
**Lactose**			
**Melbiose**			
**Mannose**	+		+
**Melezitose**			
**Inosin**			
**Mannitol**	w		
**Arbutin**			
**Sorbitol**			
**Raffinose**			
**Sorbose**			
**Rhamnose**			
**Tagatose**			
**Amygdalin**		+	
**Gluconate**			
**Salicin**			
**empty**			

Figure S1. PCR detection of the 33.8 kbp deletion present in *Lactococcus cremoris* MG1614.

Primers FDF and FDR (Table S1) flaking the deletion were used in conventional PCR. Only when a deletion is present, a 500-bp fragment could be amplified. DNA templates were extracted from several *L. cremoris* strains related to MG1614 and D1-20 (in red). *L. cremoris* MG1363 is a plasmid-cured and prophage-free derivative of strain NCDO712 and is the ancestor of MG1614; D1 and D1-20 are Lcn972R mutants evolved from MG1614; MG1614.2 is MG1614 transformed with the plasmid encoding Lcn972 biosynthesis pBL1, and NZ9000 is another derivative of MG1363 suitable for nisin-inducible gene expression.

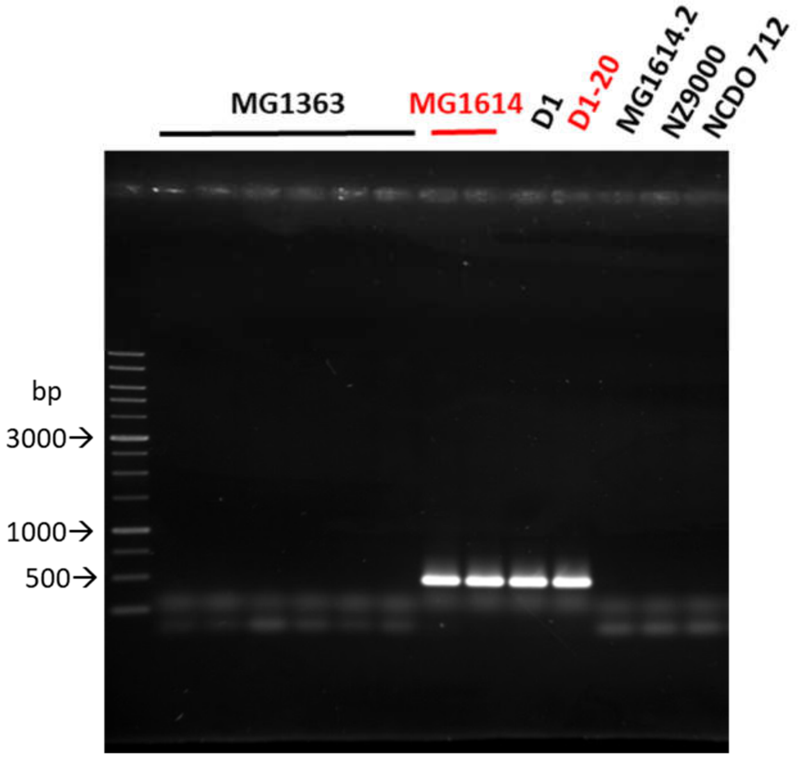

Figure S2. Presence of pinpoint colonies in samples taken at the end of the evolution experiment. *L. cremoris* MG1614 was grown in GM17 with 1,280 AU/mL Lcn972 and plated on GM17. 7A, 7B: biological replicates. M7A: representative GM17 plate from the control experiment without Lcn972.

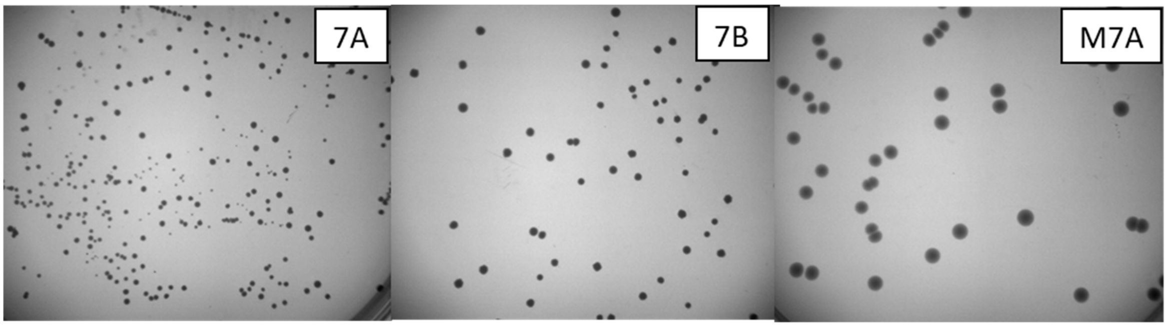

Supplement: Supplementary file 1 [file microorganisms-11-00501-s001.zip › microorganisms-2218669-supplementary.pdf]
